# Dipeptide Nitrile CD34 with Curcumin: A New Improved Combination Strategy to Synergistically Inhibit Rhodesain of *Trypanosoma brucei rhodesiense*

**DOI:** 10.3390/ijms24108477

**Published:** 2023-05-09

**Authors:** Carla Di Chio, Santo Previti, Noemi Totaro, Fabiola De Luca, Alessandro Allegra, Tanja Schirmeister, Maria Zappalà, Roberta Ettari

**Affiliations:** 1 Department of Chemical, Biological, Pharmaceutical, and Environmental Sciences, University of Messina, Viale Ferdinando Stagno d’Alcontres 31, 98166 Messina, Italy; carla.dichio@unime.it (C.D.C.); spreviti@unime.it (S.P.); noemitotaro97@gmail.com (N.T.); fabiola.deluca@studenti.unime.it (F.D.L.); mzappala@unime.it (M.Z.); 2 Department of Clinical and Experimental Medicine, University of Messina, Via C. Valeria, 98125 Messina, Italy; 3 Department of Human Pathology in Adulthood and Childhood “Gaetano Barresi”, University of Messina, Via Consolare Valeria 1, 98125 Messina, Italy; aallegra@unime.it; 4 Institute of Pharmaceutical and Biomedical Sciences, University of Mainz, Staudingerweg 5, 55128 Mainz, Germany; schirmei@uni-mainz.de

**Keywords:** cysteine protease, rhodesain inhibitors, dipeptide nitrile, curcumin, combination studies

## Abstract

Rhodesain is the main cysteine protease of *Trypanosoma brucei rhodesiense*, the parasite causing the acute lethal form of Human African Trypanosomiasis. Starting from the dipeptide nitrile **CD24**, the further introduction of a fluorine atom in the *meta* position of the phenyl ring spanning in the P3 site and the switch of the P2 leucine with a phenylalanine led to **CD34**, a synthetic inhibitor that shows a nanomolar binding affinity towards rhodesain (*K*_i_ = 27 nM) and an improved target selectivity with respect to the parent dipeptide nitrile **CD24**. In the present work, following the Chou and Talalay method, we carried out a combination study of **CD34** with curcumin, a nutraceutical obtained from *Curcuma longa L.* Starting from an affected fraction (f_a_) of rhodesain inhibition of 0.5 (i.e., the IC_50_), we observed an initial moderate synergistic action, which became a synergism for f_a_ values ranging from 0.6 to 0.7 (i.e., 60–70% inhibition of the trypanosomal protease). Interestingly, at 80–90% inhibition of rhodesain proteolytic activity, we observed a strong synergism, resulting in 100% enzyme inhibition. Overall, in addition to the improved target selectivity of **CD34** with respect to **CD24**, the combination of **CD34** + curcumin resulted in an increased synergistic action with respect to **CD24** + curcumin, thus suggesting that it is desirable to use **CD34** and curcumin in combination.

## 1. Introduction

Human African Trypanosomiasis (HAT) is a parasitic disease widespread in sub-Saharan Africa, where it represents an important death cause [1]. Two subspecies of Trypanosoma are able to induce HAT: *T. brucei gambiense*, typical of western and central Africa and responsible for the chronic form of HAT, and *T. b. rhodesiense*, widespread in eastern and southern Africa and able to induce the acute form of HAT, which has a higher mortality rate [2].

HAT is characterized by two main stages: in the first stage, also known as the hemolymphatic stage, a bloodstream invasion by the parasite induces fever and muscle aches; if untreated, the first stage can evolve into the neurological stage, in which the penetration of the central nervous system by the *Trypanosoma* induces neurological disturbances, sleep disorders, and finally death [3].

Current HAT therapy is based on four dated drugs: suramin and pentamidine, which are active in the hemolymphatic stage, and melarsoprol and eflornithine, which are used in the neurological stage. However, melarsoprol is highly toxic because it causes encephalopathy in 5–10% of treated patients [4], while eflornithine, despite its low toxicity, is active only against *T. b. gambiense* [5].

Currently, the first-line treatment of the *gambiense* HAT is based on the Nifurtimox–Eflornithine Combination Therapy (NECT), where nifurtimox, a 5-nitrofuran approved for Chagas disease, is used off-label [6]. Recently, fexinidazole, a new orally administered molecule, was introduced in therapy; however, similarly to other drugs, it has been approved only for the *gambiense* form of HAT [7,8]. In the present scenario, there is a dire need to focus the drug discovery process on new targets that will allow the identification of novel agents also active against the lethal rhodesiense form of HAT. 

In this context, rhodesain, the main cysteine protease of *T. b. rhodesiense*, is considered one of the most promising targets for the identification of novel broad-spectrum agents because of its key roles in the life cycle of the parasite [9,10,11].

Rhodesain’s importance is due to its numerous functions: (a) it allows the parasite to cross the blood–brain barrier of the human host, thus leading to the neurological stage of HAT [12]; (b) it takes part in the turnover of variant surface glycoproteins of the trypanosome coat and the degradation of host immunoglobulins, thus eluding the host’s immunoglobulins [13,14]; (c) lastly, it is involved in the degradation of intracellularly transported host proteins and parasite proteins [15]. For all these reasons, rhodesain is currently considered an important target for HAT treatment [9,10].

In this drug discovery area, our research team has been involved in the development of novel rhodesain inhibitors in the last few years [16,17,18,19,20,21,22,23,24,25,26,27,28].

**Figure 1:**
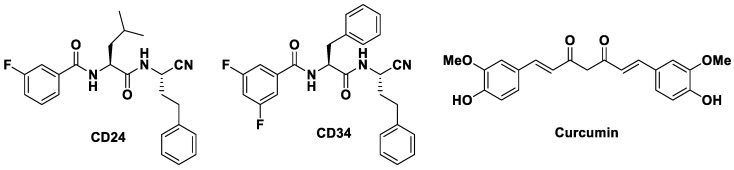
Structures of **CD24**, **CD34**, and curcumin.

Recently, we developed a new class of dipeptide nitrile able to react with the catalytic cysteine of rhodesain by Pinner reaction, giving a reversible thiomidate adduct, thus identifying a novel lead compound, i.e., the dipeptide nitrile **CD24**, which showed a nanomolar binding affinity towards rhodesain (*K*_i_ = 16 nM), coupled to a good antiparasitic activity (i.e., EC_50_ = 10.1 ± 0.5 µM) [29]. Considering our know-how in drug combinations [30,31,32], we recently carried out a combination study on the lead compound **CD24** with curcumin [33] (Figure 1), a potent multitarget nutraceutical extracted from *Curcuma longa* L., which we previously demonstrated to inhibit rhodesain in a non-competitive manner [31]. Following the rules of Chou and Talalay [34,35], we previously obtained an additive effect at the IC_50_ for this combination. It became a slight-to-moderate synergism at 60–70% of the effect, and finally a synergism from 80% to 100% of rhodesain inhibition [30].

In the present study, we developed a novel dipeptide nitrile **CD34** (Figure 1), 3,5-difluoro-substituted on the P3 aromatic nucleus. In our previous studies, we found that the incorporation of a difluoro phenyl ring at the P3 site resulted in more effective selectivity towards rhodesain with respect to the mono-fluoro-substitution [18]. Concerning the P2 site, the leucine present in **CD24** was switched with a phenylalanine in analogy to **K11777**, one of the most potent rhodesain inhibitors, with whom rhodesain has been co-crystallized (Protein Data Bank ID: 2P7U) [36].

Overall, the new fluorinated dipeptide nitrile was synthesized with the aim of investigating the reactivity of the novel inhibitor, the selectivity towards the target protease, and the possibility of using it in combination with curcumin in order to reduce the toxicity for the human host by reducing the individual dose [37]. The results of this investigation will now be presented and discussed. 

## 2. Results and Discussion

### 2.1. Chemistry

**Scheme 1:**
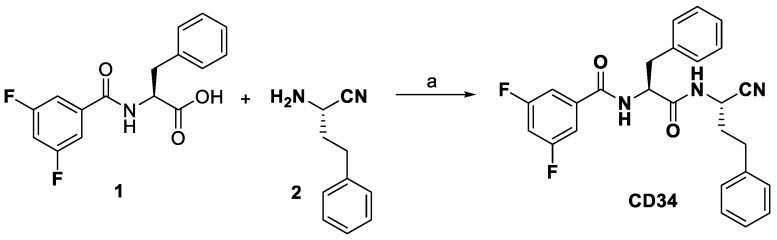
Reagents and conditions: (a) EDCI, HOBt, DIPEA, dry DCM, 0 °C to rt., on.

The dipeptide nitrile **CD34** was obtained by coupling reactions between the carboxylic acid **1** and amine **2** (Scheme 1), using ECDI and HOBt as coupling reagents, where acid **1** mimics the P3-P2 fragment while amine **2** represents the P1 synthon, both synthesized in accordance with our established synthetic procedure [19,29]. 

### 2.2. Biological Evaluation

#### 2.2.1. Inhibition of Rhodesain and Target Selectivity

**CD34** was tested against recombinant rhodesain by using Cbz-Phe-Arg-AMC as a fluorogenic substrate. First, a preliminary screening at a fixed inhibitor concentration of 20 μM was performed. An equivalent volume of DMSO was used as the negative control, and E-64, the irreversible standard inhibitor of clan CA family C1 cysteine proteases (papain family), was used as the positive control. Since **CD34** showed full inhibition of rhodesain at 20 μM, Tian continuous assays were performed at seven different concentrations, in the range 0.001–20 µM (from minimal to full enzyme inhibition), to determine the dissociation constant of the non-covalent complex enzyme–inhibitor *K*_i_ (nM) (Table 1), since **CD34** reversibly inhibited rhodesain. 

An analysis of the obtained data clearly established that both **CD24** and **CD34** inhibited rhodesain at the nanomolar level (Table 1), with a slight increase in activity for **CD24**. However, when both **CD24** and **CD34** were tested against human cathepsin L to check the selectivity of the two dipeptide nitriles towards the trypanosomal protease, **CD34** showed an improved selectivity with respect to **CD24** (SI = 7.26 vs. 2.12 for **CD34** and **CD24**, respectively). 

#### 2.2.2. Calculation of the Combination Index 

**Figure 2:**
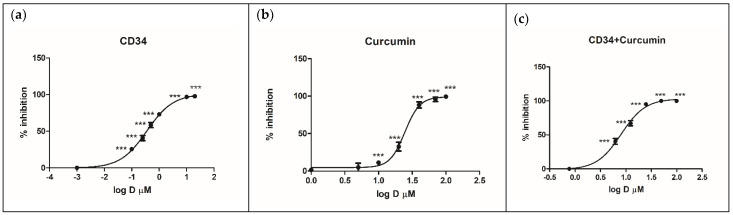
Dose-response curves for rhodesain inhibition by **CD34** (**a**), curcumin (**b**), and **CD34** + curcumin in combination (**c**). Each experiment was performed twice, each in duplicate, with *** *p* < 0.001 vs. no inhibitor.

For the calculation of the combination index (*CI*), curcumin was also tested against rhodesain in the range 1–100 µM and the IC_50_ values were obtained using GraphPad Prism 5.0.3 (GraphPad software Inc., San Diego, California) from dose-response curves as shown in Figure 2 (0.35 ± 0.035 µM for **CD34** and 24.5 ± 1.74 µM for curcumin). 

In a subsequent experiment, six data points were selected for both compounds (1/32 × IC_50_, 1/4 × IC_50_, 1/2 × IC_50_, IC_50_, 2 × IC_50_, and 4 × IC_50_, Table 2) with the aim of evaluating if a synergistic, additive, or antagonistic effect occurs in the combination study of the two inhibitors. In this assay, the combination of **CD34** and curcumin (molar ratio 1:70) provided an IC_50_ value of 8.06 ± 0.68 µM.

**Figure 3:**
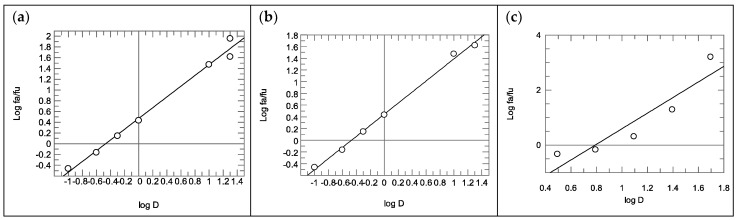
Median Effect Plot for **CD34** (**a**), curcumin (**b**), and **CD34**+ curcumin in combination (molar ratio 1:70) (**c**). D is the dose, and f_a_ and f_u_ the dose-affected and dose-unaffected fractions of rhodesain activity, respectively.

We then converted each dose-response curve into a Median Effect Plot, which was obtained by plotting the log (f_a_/f_u_) on the *y*-axis vs. the log (D) on the *x*-axis (Figure 3). In the median effect plot, the maximum response corresponds to 1 instead of 100 in the dose-response curve. Therefore, f_a_ + f_u_ = 1, where f_a_ corresponds to the “affected fraction”, i.e., the percentage of enzyme that has been inhibited, while f_u_ is the unaffected fraction, i.e., the residual enzyme activity. The slope of the straight line of each Median Effect Plot is the “m value”. In detail, **CD34** had an m_1_ value of 1.0006, curcumin had an m_2_ value of 0.9455, and the combination assay had an m_1,2_ value of 2.8377 with a **CD34**/curcumin molar ratio of 1:70. 

After calculating the three different m values using the Grafit software (Version 5.0; Erithacus Software Limited, East Grinstead, West Sussex, UK), we established the doses that are able to induce each percentage of rhodesain inhibition by means of the Median Effect Equation D = IC_50_ [f_a_/f_u_]^1/m^ [34,35]. 

With the aim of determining the inhibitory effect given by the combination of **CD34** and curcumin, we used the Chou–Talalay method to evaluate the multiple drug effects [34,35]. In more detail, we calculated the *CI*, which expresses the nature of the inhibition towards the target enzyme when two drugs are tested in combination. In particular, it is well known that *CI* > 1, *CI* = 1, and *CI* < 1 generally correspond to antagonistic, additive, and synergistic effects, respectively [34,35]. The *CI* for mutually non-exclusive drugs that act independently was calculated as follows:

*CI* = [(*D*)_1_/(*IC*_50_)_1_] + [(*D*)_2_/(*IC*_50_)_2_] + [(*D*)_1_(*D*)_2_]/[(*IC*_50_)_1_(*IC*_50_)_2_]

where (*IC*_50_)_1_ and (*IC*_50_)_2_ are obtained from the dose-response curves and *D*_1_ and *D*_2_, are the concentrations that are able to induce a specific percentage of rhodesain inhibition, as determined by the Median Effect Equation. 

**Figure 4:**
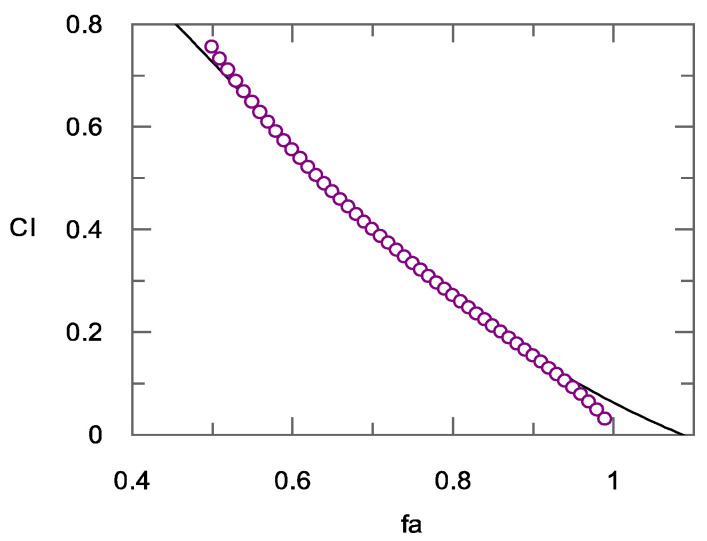
Computer-generated graphical presentation of the combination index (*CI*) vs. the fraction affected (f_a_), i.e., the effect of reduction in rhodesain activity exerted by a mixture of **CD34**–curcumin (molar ratio 1:70). The purple circles indicate the trend of the combination index.

The Grafit software was used to determine the *CI* ranging from 50% to 100% rhodesain inhibition (Figure 4). 

Starting from IC_50_ (f_a_ = 0.5), which is the first value to take into consideration when we describe the activity of a novel inhibitor, we observed an initial moderate synergistic action, which became a synergism for f_a_ ranging from 0.6 to 0.7. Interestingly, at 80–90% inhibition of rhodesain activity, a strong synergism was observed, resulting in a very strong synergism at 100% enzyme inhibition. 

## 3. Materials and Methods

### 3.1. Chemistry 

All reagents and solvents were obtained from commercial suppliers and were used without further purification. Elemental analyses were carried out on a C. Erba Model 1106 (Elemental Analyzer for C, H, and N) instrument, and the obtained results were within ±0.4% of the theoretical values. Merck silica gel 60 F254 plates were used for analytical TLC; flash column chromatography was performed on Merck silica gel (200–400 mesh). ^1^H and ^13^C NMR spectra were recorded on a Varian 500 MHz spectrometer equipped with a ONE_NMR probe, operating at 499.74 and 125.73 MHz for ^1^H and ^13^C NMR spectra, respectively. We used the residual signal of the deuterated solvent as an internal standard. Splitting patterns are described as singlet (s), doublet (d), doublet of doublet (dd), triplet (t), quartet (q), multiplet (m), or broad singlet (bs). ^1^H and ^13^C NMR chemical shifts (δ) are expressed in ppm, and coupling constants (J) are given in Hz.

### 3.2. Synthesis of Compound CD34

N-((S)-1-((S)-1-Cyano-3-phenylpropylcarbamoyl)-2-phenylethyl)-3,5-difluorobenzamide (CD34): To a solution of (S)-2-(3,5-difluorobenzamido)-3-phenylpropanoic acid **1** [19] (40 mg, 0.13 mmol) in dry DCM, EDCI (30.2 mg, 0.16 mmol), HOBt (21 mg, 0.16 mmol), and DIPEA (25.29 µL, 0.16 mmol) were added at 0 °C. After 10 min, (S)-3-amino-2-oxo-5-phenylpentanenitrile **2** [29] (14.6 mg, 0.11 mmol) was added, and the reaction mixture was stirred at room temperature overnight. After this time, the solvent was removed and the residue was dissolved in EtOAc, washed with brine, dried over Na_2_SO_4_, filtered, and concentrated in vacuo. The crude residue was purified by column chromatography using light petroleum/EtOAc 7:3 to obtain the desired coupling product, **CD34** (18 mg, 37%). Consistency: white powder; R_f_ = 0.49 (light petroleum/EtOAc 7:3); ^1^H NMR (500 MHz, CDCl_3_) (Figure S1) = 1.86–2.11 (m, 2H), 2.56–2.77 (m, 2H), 3.05–3.22 (m, 2H), 4.63–4.71 (m, 1H), 4.86–4.98 (m, 1H), 6.91–7.01 (m, 1H), 7.05–7.14 (m, 2H), and 7.15–7.35 (m, 12H) ppm; ^13^C NMR (125 MHz, CDCl_3_) (Figure S2) = δ: 31.52, 34.14, 38.62, 40.32, 55.09, 107.72 (t, J = 25.3 Hz), 110.62 (dd, J = 26.8, 3.9 Hz), 117.68, 126.88, 127.60, 128.43, 128.92, 129.02, 129.15, 129.34, 136.51 (t, J = 8.6 Hz), 139.02, 163.08 (dd, J = 251.6, 12.3 Hz), 165.42 (t, J = 3.8 Hz), and 170.67. Elemental analysis: calculated for C_26_H_23_F_2_N_3_O_2_: C 69.79, H 5.18, N 9.39; found: C 70.02, H 5.36, N 9.19.

### 3.3. Rhodesain Inhibition Assays and Calculation of the Combination Index

Curcumin was purchased from Sigma Aldrich. Since the range of active concentrations for curcumin was known by us [33], a preliminary screening with rhodesain was performed only for CD34 at 20 µM; since it inhibited rhodesain by more than 80%, it passed the initial screening and was characterized in detail. An equivalent amount of DMSO was used as negative control. Product release from substrate hydrolysis (Cbz-Phe-Arg-AMC, 10 µM) was determined continuously over a period of 10 min at room temperature. The assay buffer contained 50 mM sodium acetate, pH = 5.5, 5 mM EDTA, 200 mM NaCl, and 0.005% Brij 35 to avoid aggregation and false-positive results. The enzyme buffer contained 5 mM DTT rather than Brij 35. Inhibitor solutions were prepared from stocks of DMSO. 

CD34 and curcumin were then separately tested by Tian continuous assays, twice in duplicate in 96-well plates, for a total volume of 200 µL. In more detail, we used 0.05 µM, 0.1 µM, 0.25 µM, 0.5 µM, 1 µM, 10 µM, and 20 µM for CD34, while 1 µM, 5 µM, 10 µM, 20 µM, 40 µM, 70 µM, and 100 µM were used for curcumin. 

Fluorescence of the product AMC of the substrate hydrolyses was measured using an Infinite 200 PRO microplate reader (Tecan, Männedorf, Switzerland) at room temperature with a 380 nm excitation filter and a 460 nm emission filter. Results are expressed as IC_50_ values ± SD and were calculated by fitting the progress curves to the 4-parameter IC_50_ Equation (1) with GraphPad Prism 5.0.3 (GraphPad software Inc., San Diego, CA, USA):

(1) $$y=\frac{y_{\max}-y_{\min}}{1+{\left(\frac{\left[I\right]}{{\mathrm{IC}}_{50}}\right)}^{S}}+y_{\min}$$

where *y* (∆F/min) is the substrate hydrolysis rate; *y*_max_ is the maximum value of the dose-response curve measured at an inhibitor concentration of I = 0 µM; *y*_min_ is the minimum value obtained at high inhibitor concentrations; and *s* is the Hill coefficient.

The inhibitory constants (*K*_i_) were calculated according to the Cheng–Prusoff Equation (2):

*K*_i_ = IC_50_/(1 + [S]/*K*_m_) (2)

where S is the substrate concentration and *K*_M_ is the Michaelis–Menten constant (*K*_M_ = 0.8 µM for Cbz-Phe-Arg-AMC). 

### 3.4. Combination Index

For the calculation of the combination index of the combination CD34 + curcumin, 6 data points were used: 1/32 × IC_50F1+F2,_ 1/4 × IC_50F1+F2,_ 1/2 × IC_50F1+F2_, IC_50F1+F2,_ 2 × IC_50F1+F2,_ and 4 × IC_50F+F2_ (where F1 = CD34 and F2 = curcumin), corresponding to concentrations of 0.011 + 0.76 µM, 0.0875 + 6.125 µM, 0.175 + 12.25 µM, 0.35 + 24.5 µM, 0.7 + 49 µM, and 1.4 + 98 µM, respectively. The six combined doses were 0.7710 µM, 6.2125 µM, 12.4250 µM, 24.8500 µM, 49.7000 µM, and 99.4000 µM. 

Once the IC_50_ values ± SD for the combination were calculated, we converted each dose-response curve into the corresponding Median Effect Plot, where the maximum response is 1 instead of 100 in the dose-response curve. The fraction of enzyme that is inhibited is called the “affected fraction” (f_a_), while the fraction of enzyme that is not inhibited is called the “unaffected fraction” (f_u_), where f_a_ + f_u_ = 1. The Median Effect Plot is obtained by plotting the log (f_a_/f_u_) vs. the log (D) on the *x*-axis. This allowed us to calculate the “m value”, which represents the Hill-type coefficient, which means the sigmoidal trend (S-shaped) of the dose-response curve. 

Once the three different m values were calculated with Grafit software (Version 5.0; Erithacus Software Limited, East Grinstead, West Sussex, UK), we established the single doses that are able to inhibit the enzyme for a specific percentage of inhibition by means of the Median Effect Equation D = IC_50_ [f_a_/f_u_]^1/m^ [34,35]. 

The Chou–Talalay method was then applied to evaluate multiple drug effects [34,35]. Generally, *CI* > 1, *CI* = 1, and *CI* < 1 correspond to antagonistic, additive, and synergistic effects, respectively [34,35]. In more detail, the specific classification [38] is: very strong synergy for CI < 0.1, strong synergy for 0.1 < CI < 0.3, synergy for 0.3 < CI < 0.7, moderate synergy for 0.7 < CI < 0.85, slight synergy for 0.85 < CI < 0.9, slight antagonism for 1.1 < CI < 1.2, moderate antagonism for 1.2 < CI < 1.45, antagonism for 1.45 < CI < 3.3, strong antagonism for 3 < CI < 10, and very strong antagonism for CI > 10. 

Then, considering that CD34, in analogy to CD24 [27], acts as a competitive inhibitor and that curcumin acts as a non-competitive inhibitor, the *CI* for mutually non-exclusive drugs that act independently was calculated as follows:

*CI* = [(*D*)_1_/(*IC*_50_)_1_] + [(*D*)_2_/(*IC*_50_)_2_] + [(*D*)_1_(*D*)_2_] / [(*IC*_50_)_1_(*IC*_50_)_2_]

where (*IC*_50_)_1_ and (*IC*_50_)_2_ were obtained by dose-response curves, and *D*_1_ and *D*_2_ are the concentrations able to induce a specific percentage of rhodesain inhibition, as determined by the Median Effect Equation. 

### 3.5. Cathepsin L Inhibition Assays

The determination of the activity of the inhibition against human Cathepsin L (hCatL) was performed in fluorescence-based assays [39]. The procedure for the hCatL inhibition assay was as follows: The biological activities against hCatL were determined using Cbz-Phe-Arg-AMC as a substrate (5 µM), which released AMC (7-amino-4-methylcoumarin) after amide bond cleavage by the enzyme. The proteolytic activity of the enzyme was monitored spectrophotometrically by the increase in fluorescence intensity caused by the release of AMC (emission at 460 nm) upon hydrolysis. 

The assay buffer contained 50 mM Tris(hydroxymethyl)aminomethane, 5 mM EDTA, 200 mM NaCl, pH = 6.5, and 0.005% Brij 35 to avoid aggregation and false-positive results. The enzyme buffer contained 2 mM DTT rather than Brij 35. 

Inhibitor solutions were prepared from stocks of DMSO. An initial screening at an inhibitor concentration of 20 µM was performed to identify ligands (CD24 and CD34) with an inhibition of hCatL higher than 80%.

**CD34** and **CD24** were then tested separately by Tian continuous assays, twice in duplicate in 96-well plates, for a total volume of 200 µL. An equivalent amount of DMSO was used as negative control. In more detail, we used 0.001 µM, 0.01 µM, 0.05 µM, 0.1 µM, 0.5 µM, 1 µM, and 10 µM for CD34 and CD24. The residual enzyme activities were determined continuously over a period of 10 min at room temperature. 

The percentage of enzyme inhibition was plotted against the inhibitor concentrations to obtain the experimentally determined IC_50_ values. The inhibitory constants (*K*_i_) were calculated according to the Cheng–Prusoff Equation (1), with *K*_M_ = 6.5 µM for Cbz-Phe-Arg-AMC. 

### 3.6. Rhodesain Expression and Purification from P. pastoris and E. coli

The expression of rhodesain from *P. pastoris* was performed as described in [40]. For purification from *E. coli* Rosetta2 DE3 pLysS (Novagen), the *T. b. rhodesiense* rhodesain sequence (uniprot-ID.: Q95 PM0), followed by a TEV cleavage site and a hexahistidine-tag, was cloned between the NdeI and BamHI restriction sites of a pET-11a vector by Genscript using codon optimization. The plasmid was amplified in XL-10 cells. A GFP sequence was additionally introduced between the TEV site and the 6xHis-tag by Gibson assembly following extensive construct and purification optimization. The rhodesain plasmid and the GFP gene were amplified in a PCR using the primers 5′-CACCACCATCACCACCACTAAGG-3′ and 5′-GCTACCTTGAAAATACAGATTCTCCGGG-3′ for the vector and 5′-CTGTATTTTCAAGGTAGCGTGAGCAAGGGCGAGGAGC-3′ and 5′-GGTGGTGATGGTGGTGCTTGTACAGCTCGTCCATGCCG-3′ for the insert. 

### 3.7. Statistical Analyses

The statistical analysis of the data was performed using the one-way test (ANOVA) with Dunnett’s multiple comparison test, considering significant differences of *p* < 0.05 with respect to the percentage of rhodesain inhibition of curcumin, CD34, and curcumin + CD34. The analyses were performed with GraphPad Prism 5.0.3. Results were expressed as the arithmetic mean ± standard deviation (SD).

## 4. Conclusions

Besides the slightly higher activity of **CD24** compared to **CD34** (IC_50_ = 0.2 and 0.35, respectively), **CD34** showed increased target selectivity, which is fundamental considering the high degree of homology between rhodesain and human cathepsin L, both belonging to the papain family. 

If we compare the obtained values of *CI* with those obtained for the combination **CD24** + curcumin [33] (Table 3), it is possible to observe an initial additive effect at the IC_50,_ followed by a slight-to-moderate synergism at 60–70% and a synergistic action from 80% to 100% rhodesain inhibition. 

Thus, it is clear that the combination of **CD34** + curcumin resulted in a much stronger synergistic action, especially at 100% enzyme inhibition, where a very strong synergism was observed, thus assessing an improved combination strategy for **CD34** + curcumin with respect to that of **CD24** + the same nutraceutical. 

Considering that both **CD24** and **CD34** bind to the active site of rhodesain, while curcumin acts as a non-competitive inhibitor, a possible explanation for the increased synergistic action can be attributed to the different structural requirements of **CD34,** i.e., the presence of a phenyl alanine residue instead of a leucine, which would probably better establish van der Waals contacts with the adjacent lipophilic residues of the S2 pocket (M68, A138, L160, and A208). 

In contrast, the presence of the fluoro-substituted aromatic nucleus at the P3 site, in agreement with our previous studies [27], should be able to establish a parallel-displaced π–π interaction with F61 of the rhodesain S3 pocket, forming also additional charge–transfer interactions with the π-faces of G65 and G66 backbone peptide bonds. The presence of an additional fluorine atom at position 5 of the P3 aromatic nucleus, with regard to **CD24**, could further enhance the strength of these charge–transfer contacts. Overall, both the improvement in the strength of interactions with rhodesain and presumably the different disposition in the catalytic site of **CD34**, with regard to **CD24**, could favor the interaction with curcumin, which is believed to bind to an allosteric site close to the catalytic site. 

## Figures and Tables

**Table 1:** Rhodesain inhibition and target selectivity.

	K_i_ (nM) Rhodesain	K_i_ (nM) Cathepsin L	Selectivity Index (SI)
**CD24**	16 ± 1.8 [29]	34 ± 0.1	2.12
**CD34**	27 ± 2.7	196 ± 22	7.26
**E-64**	13 ± 0.2	30 ± 3	

**Table 2:** Six selected doses for the combination experiments of **CD34** + curcumin.

Cmps	1/32 × IC_50_	1/4 × IC_50_	1/2 × IC_50_	IC_50_	2 × IC_50_	4 × IC_50_
**CD34**	0.011 µM	0.0875 µM	0.175 µM	0.35 µM	0.7 µM	1.4 µM
Curcumin	0.76 µM	6.125 µM	12.25 µM	24.5 µM	49 µM	98 µM
**CD34** + Curcumin	0.011 + 0.76 µM	0.0875 + 6.125 µM	0.175 + 12.25 µM	0.35 + 24.5 µM	0.7 + 49 µM	1.4 + 98 µM

**Table 3:** Combination index (*CI*) at several fa values for the combination **CD24**+curcumin and **CD34**+curcumin.

Fraction Affected (f_a_)	% of Rhodesain Inhibition	*CI* CD24 + Curcumin	Diagnosis of Combined Effect CD24+ Curcumin [33]	*CI* CD34 + Curcumin	Diagnosis of Combined Effect CD34+ Curcumin
0.50	50%	1.08	Additive	0.75	Moderate Synergism
0.60	60%	0.93	Slight Synergism	0.55	Synergism
0.70	70%	0.81	Moderate Synergism	0.40	Synergism
0.80	80%	0.70	Synergism	0.27	Strong Synergism
0.90	90%	0.59	Synergism	0.15	Strong Synergism
1	100%	0.45	Synergism	0.03	Very strong Synergism

## Data Availability

Not applicable.

## Supplementary Materials

The supporting information can be downloaded at: https://www.mdpi.com/article/10.3390/ijms24108477/s1.

## Sample Availability

Samples of the compound **CD34** are available from the authors.

## References

[1] World Health Organization Human African Trypanosomiasis (Sleeping Sickness). https://www.who.int/trypanosomiasis_african/en/.

[2] Büscher P., Cecchi G., Jamonneau V., Priotto G. (2017). Human African Trypanosomiasis. Lancet.

[3] Kennedy P.G.E., Rodgers J. (2019). Clinical and neuropathogenetic aspects of Human African Trypanosomiasis. Front. Immunol..

[4] Seixas J., Atouguia J., Josenando T., Vatunga G., Bilenge C.M.M., Lutumba P., Burri C. (2020). Clinical study on the melarsoprol-related encephalopathic syndrome: Risk factors and HLA association. Trop. Med. Infect. Dis..

[5] De Koning H.P. (2020). The drugs of sleeping sickness: Their mechanisms of action and resistance, and a brief history. Trop. Med. Infect. Dis..

[6] Kansiime F., Adibaku S., Wamboga C., Idi F., Kato C.D., Yamuah L., Vaillant M., Kioy D., Olliaro P., Matovu E. (2018). A multicentre, randomised, non-inferiority clinical trial comparing a nifurtimox-eflornithine combination to standard eflornithine monotherapy for late stage Trypanosoma brucei gambiense human African trypanosomiasis in Uganda. Parasit. Vectors.

[7] Deeks E.D. (2019). Fexinidazole: First global approval. Drugs.

[8] CHMP Recommends First Oral-Only Treatment for Sleeping Sickness. https://www.ema.europa.eu/en/news/chmp-recommends-first-oral-only-treatment-sleeping-sickness.

[9] Ettari R., Previti S., Tamborini L., Cullia G., Grasso S., Zappalà M. (2016). The inhibition of cysteine proteases rhodesain and TbCatB: A valuable approach to treat Human African Trypanosomiasis. Mini Rev. Med. Chem..

[10] Ettari R., Tamborini L., Angelo I.C., Micale N., Pinto A., De Micheli C., Conti P. (2013). Inhibition of rhodesain as a novel therapeutic modality for human African trypanosomiasis. J. Med. Chem..

[11] Previti S., Di Chio C., Ettari R., Zappalà M. (2022). Dual inhibition of parasitic targets: A valuable strategy to treat malaria and neglected tropical diseases. Curr. Med. Chem..

[12] Nikolskaia O.V., de Lima A.A.P., Kim Y.V., Lonsdale-Eccles J.D., Fukuma T., Scharfstein J., Grab D.J. (2006). Blood-brain barrier traversal by African trypanosomes requires calcium signaling induced by parasite cysteine protease. J. Clin. Investig..

[13] Barry J.D., McCulloch R. (2001). Antigenic variation in trypanosomes: Enhanced phenotypic variation in a eukaryotic parasite. Adv. Parasitol..

[14] Lalmanach G., Boulange A., Serveau C., Lecaille F., Scharfstein J., Gauthier F., Authie E. (2002). Congopain from Trypanosoma congolense: Drug target and vaccine candidate. Biol. Chem..

[15] Caffrey C.R., Hansell E., Lucas K.D., Brinen L.S., Alvarez Hernandez A., Cheng J., Gwaltney S.L., Roush W.R., Stierhof Y.D., Bogyo M. (2001). Active site mapping, biochemical properties and subcellular localization of rhodesain, the major cysteine protease of *Trypanosoma brucei rhodesiense*. Mol. Biochem. Parasitol..

[16] Previti S., Ettari R., Calcaterra E., Di Chio C., Ravichandran R., Zimmer C., Hammerschmidt S., Wagner A., Cosconati S., Schirmeister T. (2022). Development of urea bond-containing Michael acceptors as antitrypanosomal agents targeting rhodesain. ACS Med. Chem. Lett..

[17] Previti S., Ettari R., Di Chio C., Ravichandran R., Bogacz M., Hellmich U.A., Schirmeister T., Cosconati S., Zappalà M. (2022). Development of reduced peptide bond pseudopeptide Michael acceptors for the treatment of Human African Trypanosomiasis. Molecules.

[18] Maiorana S., Ettari R., Previti S., Amendola G., Wagner A., Cosconati S., Hellmich U.A., Schirmeister T., Zappalà M. (2020). Peptidyl vinyl ketone irreversible inhibitors of rhodesain: Modifications of the P2 fragment. ChemMedChem.

[19] Ettari R., Previti S., Maiorana S., Amendola G., Wagner A., Cosconati S., Schirmeister T., Hellmich U.A., Zappalà M. (2019). Optimization strategy of novel peptide-based Michael acceptors for the treatment of Human African Trypanosomiasis. J. Med. Chem..

[20] Previti S., Ettari R., Cosconati S., Amendola G., Chouchene K., Wagner A., Hellmich U.A., Ulrich K., Krauth-Siegel R.L., Wich P.R. (2017). Development of novel peptide-based Michael acceptors targeting rhodesain and falcipain-2 for the treatment of Neglected Tropical Diseases (NTDs). J. Med. Chem..

[21] Ettari R., Previti S., Cosconati S., Maiorana S., Schirmeister T., Grasso S., Zappalà M. (2016). Development of novel 1,4-benzodiazepine-based Michael acceptors as antitrypanosomal agents. Bioorg. Med. Chem. Lett..

[22] Ettari R., Previti S., Cosconati S., Kesselring J., Schirmeister T., Grasso S., Zappalà M. (2016). Synthesis and biological evaluation of novel peptidomimetics as rhodesain inhibitors. J. Enzyme Inhib. Med. Chem..

[23] Ettari R., Pinto A., Previti S., Tamborini L., Angelo I.C., La Pietra V., Marinelli L., Novellino E., Schirmeister T., Zappalà M. (2015). Development of novel dipeptide-like rhodesain inhibitors containing the 3-bromoisoxazoline warhead in a constrained conformation. Bioorg. Med. Chem..

[24] Ettari R., Pinto A., Tamborini L., Angelo I.C., Grasso S., Zappalà M., Capodicasa N., Yzeiraj L., Gruber E., Aminake M.N. (2014). Synthesis and biological evaluation of papain-family cathepsin L-like cysteine protease inhibitors containing a 1,4-benzodiazepine scaffold as antiprotozoal agents. ChemMedChem.

[25] Ettari R., Tamborini L., Angelo I.C., Grasso S., Schirmeister T., Lo Presti L., De Micheli C., Pinto A., Conti P. (2013). Development of rhodesain inhibitors with a 3-bromoisoxazoline warhead. ChemMedChem.

[26] Ettari R., Micale N., Grazioso G., Bova F., Schirmeister T., Grasso S., Zappalà M. (2012). Synthesis and molecular modeling studies of derivatives of a highly potent peptidomimetic vinyl ester as falcipain-2 inhibitors. ChemMedChem.

[27] Ettari R., Zappalà M., Micale N., Schirmeister T., Gelhaus C., Leippe M., Evers A., Grasso S. (2010). Synthesis of novel peptidomimetics as inhibitors of protozoan cysteine proteases falcipain-2 and rhodesain. Eur. J. Med. Chem..

[28] Bova F., Ettari R., Micale N., Carnovale C., Schirmeister T., Gelhaus C., Leippe M., Grasso S., Zappalà M. (2010). Constrained peptidomimetics as antiplasmodial falcipain-2 inhibitors. Bioorg. Med. Chem..

[29] Di Chio C., Previti S., Amendola G., Ravichandran R., Wagner A., Cosconati S., Hellmich U.A., Schirmeister T., Zappalà M., Ettari R. (2022). Development of novel dipeptide nitriles as inhibitors of rhodesain of *Trypanosoma brucei rhodesiense*. Eur. J. Med. Chem..

[30] Di Chio C., Previti S., De Luca F., Allegra A., Zappalà M., Ettari R. (2021). Drug combination studies of PS-1 and quercetin against rhodesain of Trypanosoma brucei rhodesiense. Nat. Prod. Res..

[31] Ettari R., Previti S., Di Chio C., Maiorana S., Allegra A., Schirmeister T., Zappalà M. (2020). Drug synergism: Studies of combination of RK-52 and curcumin against rhodesain of *Trypanosoma brucei rhodesiense*. ACS Med. Chem. Lett..

[32] Ettari R., Previti S., Maiorana S., Allegra A., Schirmeister T., Grasso S., Zappalà M. (2019). Drug combination studies of curcumin and genistein against rhodesain of Trypanosoma brucei rhodesiense. Nat. Prod. Res..

[33] Di Chio C., Previti S., De Luca F., Bogacz M., Zimmer C., Wagner A., Schirmeister T., Zappalà M., Ettari R. (2022). Drug combination studies of the dipeptide nitrile CD24 with curcumin: A new strategy to synergistically inhibit rhodesain of Trypanosoma brucei rhodesiense. Int. J. Mol. Sci..

[34] Chou T.C. (2010). Drug combination studies and their synergy quantification using the Chou-Talalay method. Cancer Res..

[35] Chou T.C., Talalay P. (1984). Quantitative analysis of dose-effect relationships: The combined effects of multiple drugs or enzyme inhibitors. Adv. Enzym. Regul..

[36] Kerr I.D., Wu P., Marion-Tsukamaki R., Mackey Z.B., Brinen L.S. (2010). Crystal Structures of TbCatB and rhodesain, potential chemotherapeutic targets and major cysteine proteases of Trypanosoma brucei. PLoS Negl. Trop. Dis..

[37] Pourkavoos N. (2012). Unique risks, benefits, and challenges of developing drug-drug combination products in a pharmaceutical industrial setting. Comb. Prod. Ther..

[38] Sisi W., Hongyong Z., Liang C., Christopher E., Chong-Xian P.A.N. (2010). Analysis of the cytotoxic activity of carboplatin and gemcitabine combination. Anticancer Res..

[39] Previti S., Ettari R., Calcaterra E., Di Maro S., Hammerschmidt S.J., Muller C., Ziebuhr J., Schirmeister T., Cosconati S., Zappalà M. (2023). Structure-based lead optimization of peptide-based vinyl methyl ketones as SARS-CoV-2 main protease inhibitors. Eur. J. Med. Chem..

[40] Johe P., Jaenicke E., Neuweiler H., Schirmeister T., Kersten C., Hellmich U.A. (2021). Structure, interdomain dynamics, and pH-dependent autoactivation of pro-rhodesain, the main lysosomal cysteine protease from African trypanosomes. J. Biol. Chem..

