# Supplementary material for: Dipeptide Nitrile CD34 with Curcumin: A New Improved Combination Strategy to Synergistically Inhibit Rhodesain of Trypanosoma brucei rhodesiense
Source: Int J Mol Sci. 2023 May 9;24(10):8477. doi: 10.3390/ijms24108477 (PMC10218348; doi:10.3390/ijms24108477)
Supplement: Supplementary file 1 [file ijms-24-08477-s001.zip › ijms-2338121-supplementary.pdf]

Supplementary material

Dipeptide nitrile CD34 with curcumin: a new improved combination strategy to synergistically inhibit rhodesain of *Trypanosoma brucei rhodesiense*

Carla Di Chio[1,2,†], Santo Previti[1, †], Noemi Totaro[1], Fabiola De Luca[1], Alessandro Allegra[3], Tanja Schirmeister[4], Maria Zappalà[1], and Roberta Ettari[1,*]

[1] Department of Chemical, Biological, Pharmaceutical, and Environmental Sciences, University of Messina, Viale Ferdinando Stagno d'Alcontres 31, 98166 Messina, Italy
[2] Department of Clinical and Experimental Medicine, University of Messina, Via C. Valeria, 98125 Messina, Italy
[3] Department of Human Pathology in Adulthood and Childhood "Gaetano Barresi", University of Messina, Via Consolare Valeria 1, 98125 Messina, Italy
[4] Institute of Pharmaceutical and Biomedical Sciences, University of Mainz, Staudingerweg 5, 55128 Mainz, Germany
* Correspondence: rettari@unime.it; Tel.: +39-090-676-6554
† These authors equally contributed to this work.

Table of contents

Figure S1. ^{1}H NMR spectrum of **CD34**.

Figure S2. ^{13}C NMR spectrum of **CD34**.

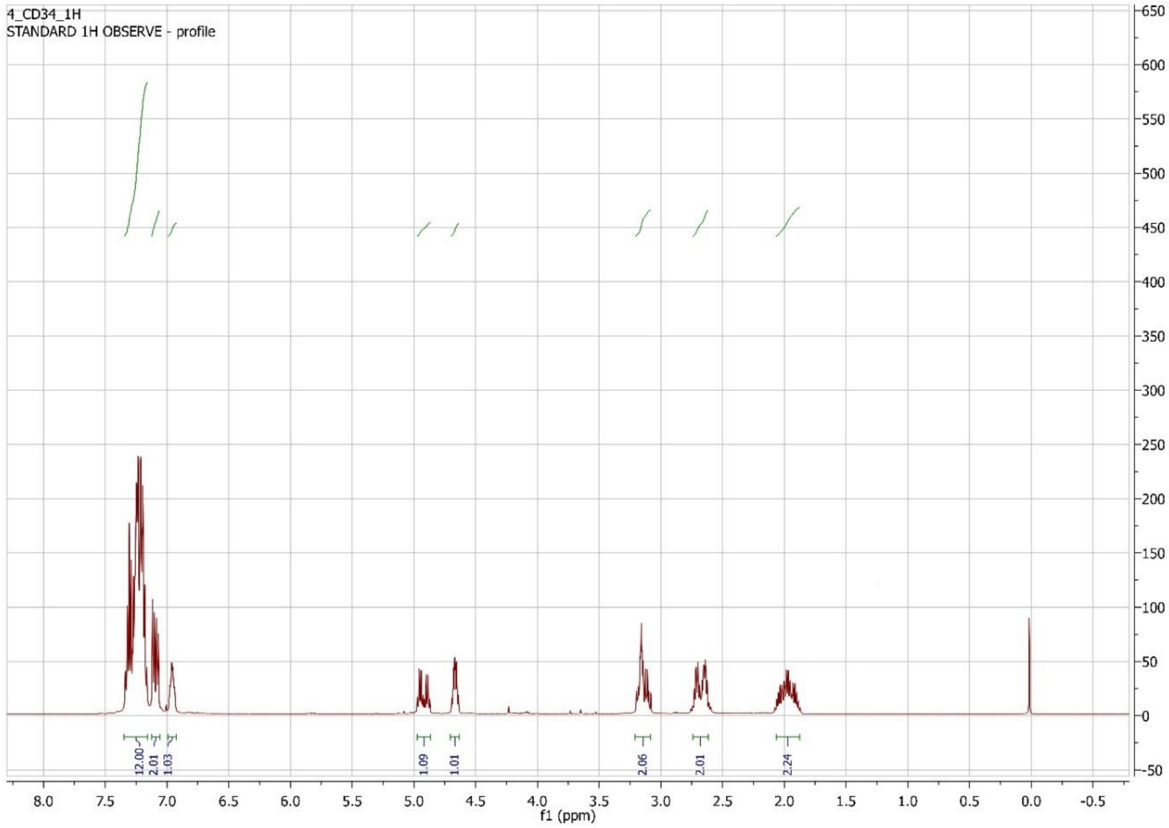

Figure S1. ^{1}H NMR spectrum of **CD34**.

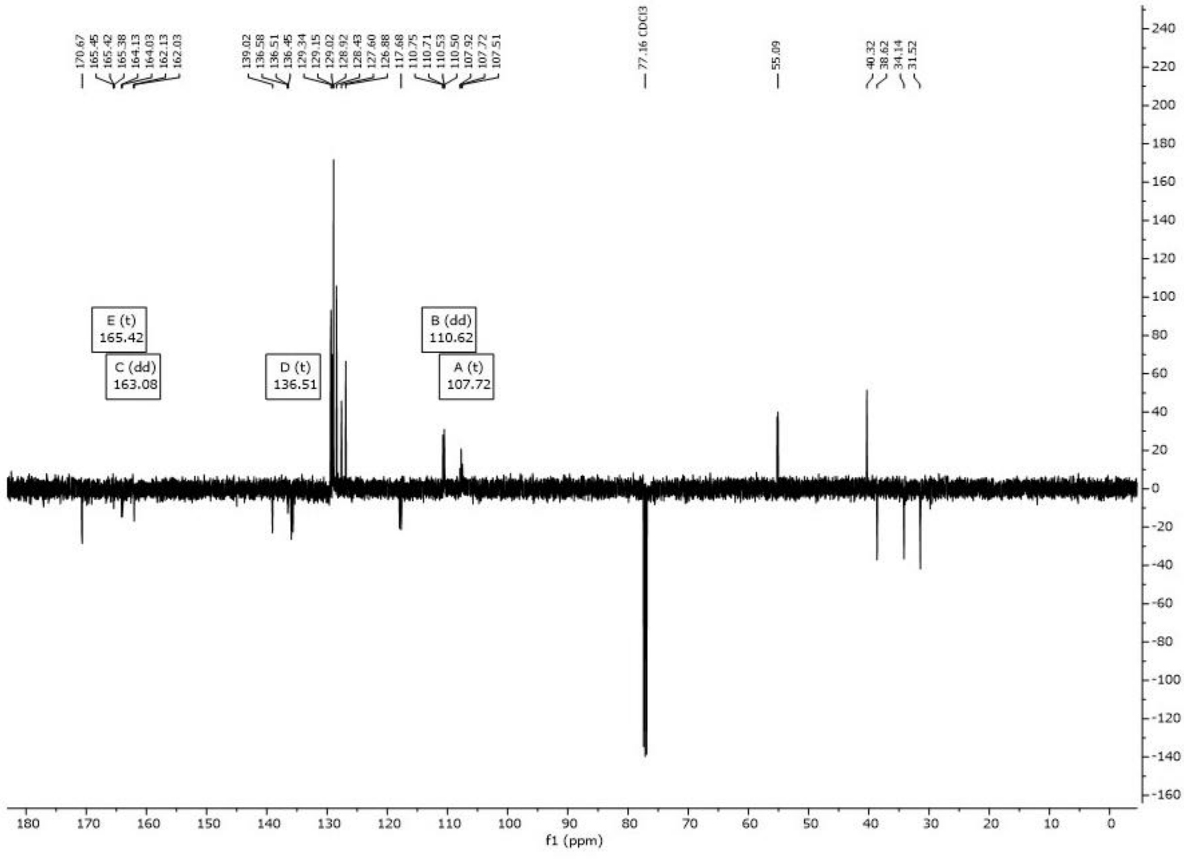

Figure S2. ^{13}C NMR spectrum of **CD34**.
